# The Predictive Potential of Elevated Serum Inflammatory Markers in Determining the Need for Intubation in CoVID-19 Patients

**DOI:** 10.2478/jccm-2021-0035

**Published:** 2021-11-13

**Authors:** Samuel Windham, Kellen Hirsch, Ryan Peterson, David Douin, Lakshmi Chauhan, Lauren Heery, Connor Fling, Nemanja Vukovic, Fernando Holguin, Shanta Zimmer, Kristine Erlandson

**Affiliations:** 1 University of Colorado Anschutz Medical Campus, Denver, CO, USA

**Keywords:** intubation, CoVID-19, SARS-CoV-2, inflammatory markers

## Abstract

**Introduction:**

The predictive potential of demographics, clinical characteristics, and inflammatory markers at admission to determine future intubation needs of hospitalised CoVID-19 patients is unknown. The study aimed to determine the predictive potential of elevated serum inflammatory markers in determining the need for intubation in CoVID-19 Patients.

**Methods:**

In a retrospective cohort study of hospitalised SARS-CoV2 positive patients, single and multivariable regression analyses were used to determine covariate effects on intubation odds, and a minimax concave penalty regularised logistic regression was used to build a predictive model. A second prospective independent cohort tested the model.

**Results:**

Systemic inflammatory markers obtained at admission were higher in patients that required subsequent intubation, and adjusted odds of intubation increased for every standard deviation above the mean for c-reactive protein (CRP) OR:2.8 (95% CI 1.8-4.5, p<0.001) and lactate dehydrogenase OR:2.1 (95% CI 1.33.3, p=0.002). A predictive model incorporating C-reactive protein, lactate dehydrogenase, and diabetes status at the time of admission predicted intubation status with an area under the curve (AUC) of 0.78 with corresponding sensitivity of 86%, specificity of 63%. This predictive model achieved an AUC of 0.83, 91% sensitivity, and 41% specificity on the validation cohort.

**Conclusion:**

In patients hospitalised with CoVID-19, elevated serum inflammatory markers measured within the first twenty-four hours of admission are associated with an increased need for intubation. Additionally, a model of C-reactive protein, lactate dehydrogenase, and the presence of diabetes may play a predictive role in determining the future need for intubation.

## Introduction

Severe acute respiratory syndrome coronavirus 2 (SARS-CoV-2), and its concomitant clinical disease coronavirus disease 2019 (CoVID-19), is an ongoing pandemic originating in China that has quickly spread across the world. As of late October, over 42 million cases and 1.1 million deaths have been reported worldwide [1]. Similar to previous coronavirus infections, patients infected with this novel coronavirus have a variable clinical course. The severity of the disease can vary widely amongst individuals: Fifty-two per cent to eighty-two per cent of patients requiring hospital admission will require supplemental oxygen, and 24-32% will require intubation [2, 3, 4, 5, 6]. Given the variable and possibly unpredictable clinical course of many CoVID-19 patients and the potential scarcity of intubation resources worldwide, more data are needed to identify those at greater risk for intubation.

Previous studies have found higher median C-reactive protein, lactate dehydrogenase, neutrophil-tolymphocyte ratio and D-dimer among hospitalised patients with severe CoVID-19 than less severe disease [7,8]. Other studies have developed predictive modelling for critical illness as a term encompassing ICU admission, need for intubation, and death on admission [9,10]. Regarding triaging admitted patients, one of the essential aspects of critical illness is the need for intubation.

The study aimed to determine the predictive potential of elevated serum inflammatory markers in determining the need for intubation in patients admitted for CoVID-19, based on admission data. The null hypothesis is that mean serum inflammatory marker levels are not equal for intubated and non-intubated patients.

## Methods

A retrospective cohort study was undertaken using the records of the first 158 hospitalised patients aged 18 or older who tested positive for SARS-CoV-2 by nasopharyngeal polymerase chain reaction (PCR) at the University of Colorado Hospital in Aurora, Colorado, between March 19, 2020, and April 2, 2020. Data were collected from the electronic medical record and chart abstraction and recorded in REDCap. Patients were excluded if their records showed they had concomitant viral, bacterial, or fungal co-infections at the time of admission (n=0). The primary outcome was the need for intubation. The institution’s criteria for intubation was based on patients who could not maintain >90% oxygenation saturation on maximum settings of low-flow oxygen delivery devices. This assessment was carried out by attending staff of the intensive care unit. During the study period, positive pressure ventilation was not utilised due to initial concerns regarding aerosolisation of SARS-CoV-2. Additional outcomes included the need for vasopressor medication, prone positioning, hospital discharge, and mortality on or before April 6, 2020. The second cohort of one hundred and two patients consecutively admitted after April 2, 2020, with the same inclusion criteria, exclusion criteria, and chart data were used to validate the predictive model. The study was reviewed and approved by the Colorado Multiple Institutional Review Board as exempt research.

Categorical and continuous variables were first compared against intubation with Fisher’s Exact and Kruskal-Wallace tests, respectively. Then, D-dimer and ferritin were log-transformed. Intubation odds ratios (OR) for covariates of interest are presented as unadjusted and adjusted for a priori confounding variables: age, type 2 diabetes mellitus (DM), symptom duration, ethnicity, and body mass index (BMI). Nonlinear relationships of covariates with intubation were considered using generalised additive models via the mgcv R package [11]. (Supplemental online material - Figure A) Time to intubation by C-reactive protein was illustrated and tested via Kaplan-Meier curves and the log-rank test. Multiple imputations by chained equations via the mice R package [12] was used. In addition, missing data were modelled using random forests and imputed 20 times, and results from the primary analyses were computed and pooled across all imputed data sets.

The minimax concave penalty (MCP) regularised logistic regression [13] was employed to build a sparse predictive model using the covariates and confounders. This procedure fits a series of increasingly saturated models, where saturation is varied using MCP’s tuning parameter λ. A final MCP model indicated covariates most predictive of intubation. The MCP model was tuned via 10-fold cross-validation (CV) using the ncvreg R package [14]. The optimal model, selected by minimising CV deviance, is presented as a probability formula predicting intubation. The approximate formula was estimated with an MCP-regularized linear probability model. The receiver operating characteristic (ROC) curve and accompanying area under the curve, sensitivity, and specificity were calculated using CV predictions of our optimally tuned models (logistics and linear approximation). The model fitting for each imputed data set was performed to obtain coefficients and took the median coefficient estimate across all imputed data sets to account for missing data. Finally, single imputation was used to represent the figures and CV estimates. The data from the second cohort of 102 patients were plotted on a ROC curve using the predictive model for validating its accuracy. The Significance level was set at 0.05 for all relevant statistical analyses.

## Results

### CoVID-19 Patient Characteristics

Of the initial 158 patients admitted and tested positive for SARS-CoV-2 by nasopharyngeal PCR, the mean age (SD) was 56 years (16.8), Table 1. Fifty-one per cent of patients were male, and 49% were female. Eighty-one per cent of patients identified as racial or ethnic minorities. The most prevalent comorbidities were hypertension (56%), obesity (54%) and *diabetes mellitus* (30%). In addition, patients most commonly presented with cough (82%), dyspnea (75%), and fever (74%). The average duration in days (SD) of symptoms before admission was 6.6 (4.6). Chest x-rays showed bilateral infiltrates in 102 patients (65%), unilateral infiltrates in 28 patients (18%), and no evidence of acute pulmonary process in 11% of patients.

**Table 1:** Baseline Characteristics of Hospitalised CoVID-19 Patients

	Overall (N=158)
Clinical Characteristics	
Age	56.2 (16.8)
BMI	31.3 (8.0)
Male	81 (51.3%)
Active Tobacco Use	9 (5.8%)
White, non-Hispanic	30 (19.5%)
Black	53 (34.4%)
Hispanic	45 (29.2%)
Asian	11 (7.1%)
Other	15 (9.7%)
Diabetes	47 (30.3%)
Hypertension	87 (56.1%)
COPD	11 (7.1%)
Creatinine >2 mg/dL on Admission	4 (2.5%)
Cirrhosis	3 (1.9%)
Coronary Artery Disease	18 (11.6%)
Active Cancer	11 (7.1%)
Immunosuppressed	10 (6.5%)

**Presenting Symptoms**	
Subjective Fever	111 (74.0%)
Cough	126 (82.4)
Diarrhoea	40 (26.8%)
Nausea/Vomiting	42 (28.2%)
Myalgia	34 (22.8%)
Dyspnoea	116 (75.3%)
Duration of Symptoms	6.7 (4.6)

Chest X-Ray Findings*	
Unilateral Infiltrate	28 (17.8%)
Bilateral Infiltrate	102 (65%)
Other Findings	28 (17.8%)

**Clinical Course**	
Intubation Indicated	64 (40.5%)
Vasopressors Indicated	42 (26.6%)
Prone Positioned	24 (15.2%)
Deceased	4 (2.5%)
Discharge	55 (36.9%)

Data are reported as number (%) or mean (standard deviation). * Data collected within 24 hours of hospital admission. BMI = body mass index, COPD = chronic obstructive pulmonary disease.

### Hospital Course

Table 1 shows that 55 (37%) patients were discharged from the hospital without the need for invasive ventilation, vasopressors, or prone positioning on or before April 6. Sixty-four (41%) of patients required intubation, 27% required vasopressor medications, and 15% were prone positioned during intubation. Four patients (2.5%) died.

### Characteristics of Intubated Patients

Oxygen requirements, clinical characteristics, and P: F ratio at an interval of eight hours are shown in Table 2. The average SOFA score on intubation was 4.5 (SD 2.1), patients were intubated for 11.9 (SD 8.5) days, and one patient was put on non-invasive ventilation before intubation. The average P: F ratio on intubation was 146.3 (SD 84.1) in the initial eight hours from intubation and 219.9 (SD 81.4) at 72 hours from intubation.

**Table 2:** Characteristics of Intubated CoVID-19 Patients

	Overall (N=64)
**Clinical Characteristics**	
Oxygen Requirement on Admission in Litres of Oxygen	5.6 (13.2)
Highest Oxygen Requirement Stable >1 Hour within 24 Hours of Admission in Litres of Oxygen	5.0 (4.1)
Use of Non-Invasive Ventilation Before Intubation	1 (1.6%)
Oxygen Requirement within one hour of Intubation in Litres of Oxygen	12.9 (12.7)
SOFA on Intubation	4.5 (2.1)
Days Intubated	11.926 (8.519)

**Pa02 to Fi02 at 8 Hour Intervals**	
P:F Ratio at 8h	146.3 (84.1)
P:F Ratio at 16h	194.9 (69.1)
P:F Ratio at 24h	204.0 (63.4)
P:F Ratio at 32h	216.8 (65.5)
P:F Ratio at 40h	216.2 (65.9)
P:F Ratio at 48h	204.7 (75.3)
P:F Ratio at 56h	212.5 (73.9)
P:F Ratio at 64h	217.0 (71.2)
P:F Ratio at 72h	219.9 (81.4)

Pa02 to (Fi02*PEEP) at 8 Hour Intervals	
P:FP Ratio at 8h	12.3 (7.4)
P:FP Ratio at 16h	17.3 (9.8)
P:FP Ratio at 24h	17.5 (8.0)
P:FP Ratio at 32h	19.1 (8.8)
P:FP Ratio at 40h	20.1 (10.8)
P:FP Ratio at 48h	18.2 (13.7)
P:FP Ratio at 56h	19.1 (9.5)
P:FP Ratio at 64h	20.6 (12.8)
P:FP Ratio at 72h	21.2 (13.0)

Data are reported as number (%) or mean (standard deviation). h = hour, Pa02 = Arterial oxygen content, Fi02 = fractional of inspired oxygen, PEEP = Positive end expiratory pressure, P:F = Pa02/ Fi02, P:FP = Pa02/(Fi02*PEEP)

### Differences between Intubated and Non-Intubated Patients

Table 3 shows no significant differences in age, body mass index (BMI), or ethnicity between patients with or without intubation. Intubated patients were more likely to have *diabetes mellitus* (43 vs 22%, p=0.012), but not other comorbidities.

**Table 3:** Characteristics of Hospitalised CoVID-19 Patients Stratified by Intubation Status

	Not Intubated (N=94)	Intubated (N=64)	N-Missing (Not intubated/ Intubated)	p-value
**Clinical Characteristics**				
Age	55.36 (17.33)	57.32 (15.90)	0/1	0.415
BMI	30.85 (7.84)	32.01 (8.2)	5/0	0.436
Male	45 (47.9%)	36 (56.2%)	NA	0.333
Active Tobacco Use	4 (4.3%)	5 (8.1%)	NA	0.485
Non-White Race and/or Hispanic	71 (76.3%)	53 (86.9%)	NA	0.145
Ethnicity				
Diabetes	21 (22.3%)	26 (42.6%)	NA	0.012
Hypertension	52 (55.3%)	35 (57.4%)	NA	0.869
COPD	6 (6.4%)	5 (8.2%)	NA	0.753
Creatinine >2 mg/dL on Admission	1 (1.1%)	3 (4.8%)	NA	0.303
Cirrhosis	1 (1.1%)	2 (3.3%)	NA	0.562
Coronary Artery Disease	12 (12.8%)	6 (9.8%)	NA	0.620
Active Cancer	6 (6.4%)	5 (8.2%)	NA	0.753
Immunosuppressed	8 (8.6%)	2 (3.3%)	NA	0.317

**Presenting Symptoms**				
Subjective Fever	72 (77.4%)	39 (68.4%)	NA	0.253
Cough	75 (79.8%)	51 (86.4%)	NA	0.385
Diarrhoea	30 (32.3%)	10 (17.9%)	NA	0.059
Nausea/Vomiting	33 (35.5%)	9 (16.1%)	NA	0.014
Myalgia	20 (21.5%)	14 (25%)	NA	0.688
Dyspnoea	74 (78.7%)	42 (70%)	NA	0.253
Duration of Symptoms	6.7 (4.53)	6.57 (4.81)	1/6	0.796

**Chest X-Ray Findings** ^*^				
Clear	12 (13.8%)	4 (6.3%)	NA	0.192
Unilateral Infiltrate	21 (22.3%)	7 (11.1%)	NA	0.090
Bilateral Infiltrate	51 (54.3%)	51 (81%)	NA	<0.001
Pleural Effusion	0 (0%)	2 (3.2%)	NA	0.159
Other Finding	6 (6.4%)	3 (4.8%)	NA	0.741

**Laboratory Findings** ^*^				
CRP mg/L	70.34 (60.5)	142.50 (83.36)	2/6	<0.001
LDH U/L	311.53 (102.3)	415.37 (160.22)	5/13	<0.001
D-dimer FEU	1199.23 (1692.86)	4475.20 (14140.16)	15/14	0.033
Ferritin ng/mL	428.47 (426.81)	790.62 (1236.12)	7/17	0.079
Neutrophils x10^9/L	4.55 (2.11)	7.20 (3.68)	59/24	<0.001
Lymphocytes x10^9/L	3.60 (14.03)	1.08 (0.63)	59/24	0.067
NLR	4.20 (2.41)	8.33 (6.51)	59/24	<0.001

**Clinical Course**				
Deceased	2 (2.1%)	2 (3.1%)	NA	1.00
Discharged	53 (57.6%)	2 (3.5%)	NA	<0.001

Data are reported as number (%) or mean (standard deviation). * Data collected within 24 hours of hospital admission. BMI = body mass index, COPD = chronic obstructive pulmonary disease, CRP = C-reactive protein, LDH = lactate dehydrogenase, FEU = fibrinogen equivalent units, NLR = neutrophil-lymphocyte ratio. NA signifies no missing data.

Intubated patients had a similar duration of symptoms but were less likely to report nausea or vomiting (16 vs 36%, p=0.014) or diarrhoea (18 vs 32%, p=0.06). No other differences in presenting symptoms were observed between the two groups.

Intubated patients were more likely to have bilateral lung infiltrates on admission chest X-ray (81 vs 54%, p<0.001). In contrast, unilateral lung infiltrates were seen in 22% of the non-intubated group vs 11.1% of the intubated group (p=0.09).

Differences in systemic inflammatory markers among patients that did or did not require intubation are shown in Table 3. Compared to non-intubated patients, those requiring intubation had significantly higher C-reactive protein (mean=70.3 mg/L vs 142.5 mg/L, p<0.001) and lactate dehydrogenase (311.5 U/L vs 415.4 U/L, p<0.001). Ferritin, D-dimer, and the neutrophillymphocyte ratio were also significantly higher in intubated patients (p=0.08, p=0.03, p<0.001, respectively) (Figure A). Therefore the null hypothesis is upheld.

The effect of inflammatory markers and other clinical predictors were evaluated on the odds for intubation, before and after adjusting for confounding variables such as age, race or ethnicity, sex, BMI, diabetes, and symptom duration). As shown in Table 4 and Supplemental online material - Figure B, in univariate models, higher C-reactive protein, lactate dehydrogenase, D-dimer, neutrophil-lymphocyte ratio, *diabetes mellitus*, and bilateral infiltrate on chest x-ray were associated with greater odds of requiring intubation. After adjusting, the odds of intubation were significantly greater as inflammatory markers increased, per standard deviation increase in C-reactive protein (OR 2.81), lactate dehydrogenase (OR 2.10), and neutrophil-lymphocyte ratio (OR 2.21). For example, for every increase of 78 mg/L in C-reactive protein, the odds of intubation are multiplied by 2.81. We did not observe a significant effect of age, sex, ethnicity, BMI, symptom duration, ferritin, or D-dimer on the odds for intubation. Collinearity among variables was also explored (Supplemental online material - Table A).

**Table 4:** Covariate Effects on Odds of Intubation

		Unadjusted			Adjusted *	
Term	OR	95% CI	p-value	OR	95% CI	p-value
1 SD change in CRP (78.0 mg/L)	3.05	(1.9, 4.8)	<0.001	2.81	(1.8, 4.5)	<0.001
1 SD change in LDH (135.6 U/L)	2.31	(1.5, 3.6)	<0.001	2.10	(1.3, 3.3)	0.002
1 SD change in Log D-Dimer (0.94 log FEU)	1.47	(1, 2.2)	0.048	1.33	(0.8, 2.1)	0.22
1 SD change in NLR (5.4)	1.94	(1.1, 3.5)	0.031	2.21	(1.1, 4.5)	0.033
1 SD change in Log Ferritin (1.12 log ng/mL)	1.45	(1, 2.1)	0.055	1.29	(0.9, 1.9)	0.23
1 SD change in BMI (8.0 kg/m^2^)	1.15	(0.8, 1.6)	0.39	1.07	(0.7, 1.6)	0.71
1 SD change in age (16.8 years)	1.13	(0.8, 1.6)	0.45	1.15	(0.8, 1.7)	0.48
Symptom Duration	0.99	(0.9, 1.1)	0.86	0.99	(0.9, 1.1)	0.72
DM	2.52	(1.3, 5.1)	0.010	1.98	(0.9, 4.3)	0.081
Ethnicity/Race is Non-White	2.08	(0.9, 5)	0.10	1.96	(0.8, 5.1)	0.17
Bilateral Infiltrate on X-Ray	3.6	(1.7, 7.6)	0.001	3.39	(1.5, 7.6)	0.003

Note on Interpretation: For every (*_n_*) SD increase in each covariate, we expect the odds of intubation to increase by OR^(n)^ for each respective covariate. For example: after controlling for age, sex, DM, BMI, and symptom duration, for every 78 mg/L (1 SD) increase in CRP, we expect odds of intubation to increase by a factor of 2.81. For every 156 mg/L (2 SD) increase in CRP, we expect odds of intubation to increase by a factor of 2.812=7.90. * Adjusts for age, DM, symptom duration, ethnicity, and BMI. SD = standard deviation, OR = odds ratio, CI = confidence interval, CRP = C-reactive protein, LDH = lactate dehydrogenase, FEU = fibrinogen equivalent units, NLR = neutrophil-lymphocyte ratio, BMI = body mass index, DM = diabetes mellitus.

In a sensitivity analysis, the effect of each covariate on the odds for intubation for patients that were intubated at least 24 hours after admission (n=23) versus those not requiring intubation (n=94) were explored. C-reactive protein, lactate dehydrogenase, and bilateral infiltrate on chest x-ray remained significantly associated with later intubation, compared to no intubation, with an unadjusted odds ratio of 2.24 (95% CI 1.5, 3.4, p<0.001), OR 1.87 (95% CI 1.2, 2.9, p=0.006), and OR 3.37 (95% CI 1.3, 8.5, p=0.01), respectively (Supplemental online material - Table B).

### Characterisation of Patients by Serum C-reactive protein Concentration and Probability of Intubation Over Time by Serum C-reactive protein Concentration

**Fig.1:**
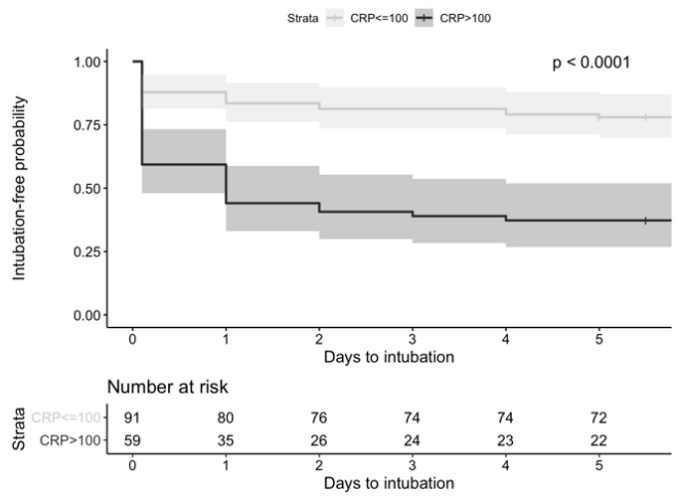
Kaplan-Meier curves for intubation-free survival of hospitalized CoVID-19 patients stratified by C-reactive protein (CRP). Patients with low CRP (<100 mg/L) have a significantly higher probability of iuntubation-free survival, as compared to patients with high CRP (≥100 mg/L) (p<0.001)

To understand the role of low versus high C-reactive protein in clinical characteristics and outcomes, patients were stratified by a C-reactive protein concentration of ≤100 mg/L versus higher than 100 mg/L (Supplemental online material - Table C). Patients with higher C-reactive protein were more likely to present with cough (p=0.027) and bilateral lung infiltrates on chest x-ray (p=0.009), while those with low C-reactive protein were more likely to present with nausea/ vomiting (p=0.022) and have a negative chest x-ray (p=0.016). No other significant differences were detected in presenting characteristics by C-reactive protein stratification. However, as shown in the Kaplan-Meier curve Figure 1, the probability of intubation over time was significantly greater in the high versus low C-reactive protein group (p<0.0001), with more than half of patients in the high C-reactive protein group intubated within the first two days of admission.

### Generating a Predictive Model for Probability of Intubation

**Fig. 2:**
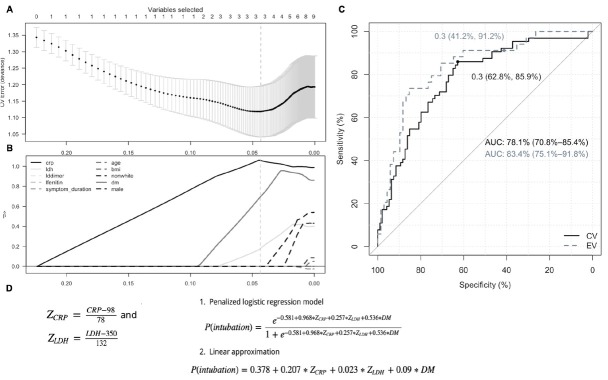
Predictive model for intubation and mechanical ventilation fit via the Minimax Concave Penalty. (A) Cross-validation deviance indicates optimal model has 3 predictors, including (B) C-reactive protein (CRP), lactate dehydrogenase (LDH), and type 2 diabetes mellitus as most predictive covariates. (C) Cross validated ROC curve of logistic model (CV) along with ROC curve on the validation cohort (EV). (D) Optimal model shown as probability formula where coviariates are centered and scaled using observed mean and standard deviation. DM signifies patient has type 2 diabetes (yes=1, no=0). Both logistic regression and linear approximation formulas presented.

Using a sparse predictive model using the covariates of interest (C-reactive protein, lactate dehydrogenase, D-dimer, ferritin, age, BMI, non-white, *diabetes mellitus*, male, symptom duration before admission, and bilateral chest x-ray infiltrates), C-reactive protein, lactate dehydrogenase and *diabetes mellitus* were selected by the model as the most predictive factors for predicting the need for intubation. These three variables correctly classified intubated status 71% of the time, corresponding to a sensitivity of 86%, specificity of 63%, positive predictive value (PPV) of 61%, negative predictive value (NPV) of 87% using a 30% threshold. The area under the ROC curve (AUC) was 78% (Figure 2).

Bilateral infiltrates on chest x-ray added marginally to the model’s predictive performance (data not shown) but was removed as it resulted in a decrease in model sensitivity. The predictive model was then applied to the separate 102 patient validation cohort data whose descriptive data are listed in Supplemental online material - Table D, which showed a sensitivity of 91%, specificity of 41%, PPV of 44%, and NPV of 90% at the 30% threshold. The prediction model diagnostic accuracy with alternative prediction thresholds are shown in Supplemental online material - Table E.

## Discussion

A retrospective cohort study characterised the demographics, comorbidities, presenting symptoms, and clinical findings of the first 158 patients admitted with positive SARS-CoV-2 at a large tertiary care institution. In addition, a variety of risk factors for intubation in these hospitalised patients, including *diabetes mellitus*, bilateral infiltrate on chest x-ray, and elevated inflammatory markers were identified.

In a predictive model, a high C-reactive protein, lactate dehydrogenase and presence of *diabetes mellitus* predicted the need for intubation, with high sensitivity and moderate specificity. These findings were replicated in a separate validation cohort, revealing a similarly high sensitivity and moderate specificity, key characteristics for a screening test where a more cautious approach is desired. The initial C-reactive protein on admission was predictive of subsequent risk for intubation.

There is a need to quickly identify and triage high-risk patients with CoVID-19 who are likely to require intubation, both for appropriate patient triage and management of limited resources [15]. The described derived model is easy to apply and utilises commonly available laboratory values that have biologically plausible links to the disease process and clinical history previously shown to be a risk factor for intubation in pneumonia [16]. It provided good sensitivity for risk for intubation when used in a cohort with many similarities, although our validation cohort did require more prone positioning and had more deceased patients despite having more patients reach discharge in the specified time frame. Given the strain on resources CoVID-19 represents, it will be critically important to identify where resources will need to be used as soon as possible. The present findings that three simple measures, high C-reactive protein, lactate dehydrogenase, and presence of *diabetes mellitus*, can quickly identify those at highest risk for intubation can augment clinical findings in identifying patients that may necessitate intensive care unit admission.

Previous studies have examined the predictive qualities of inflammatory markers in CoVID-19, although to a different degree than this study. In a retrospective study, Barrett et al. (2021), a cohort of 1123 patients with CoVID-19 were analysed for initial laboratory values and severity of illness, including death, intubation, need for renal replacement therapy, and ICU admission [17]. Univariate analysis found statistically significant, but weak links between elevated C-reactive protein and lactate dehydrogenase and the need for intubation with Spearman’s p^2^ reported as 0.090 and 0.108, respectively. A C-reactive protein of >8mg/L showed 100% sensitivity and negative predictive value for intubation when variables were dichotomised to normal or above normal. The specificity and positive predictive value were poor for C-reactive protein at 4% and 27%.

Compared to the dichotomised approach taken by Barret et al. (2021), the relationship between intubation and inflammatory markers as continuous and categorical variables in the analysis was tested. This allowed us to explore higher levels of inflammatory markers as predictors of severe disease. Ultimately, a cut-off for C-reactive protein of 100mg/L in our final predictive model, which was higher than the cut-off of 8mg/dl used in the Barrett et al. (2021) study, was used. The population studied by Barrett et al. was dissimilar to the population in the current study, with higher patients with chronic kidney disease, hypertension, coronary artery disease, and diabetes [17]. These differences in population, approach, and analysis may account for the differing predictive ability noted. Moving forward, the validity of this predictive model should be tested in large populations with different demographics and comorbidities.

In line with previous data regarding the severity of disease and gastrointestinal symptoms, gastrointestinal symptoms such as nausea, vomiting or diarrhoea were negatively correlated with the need for intubation [10]. Other studies have found no link between mortality, need for intubation, nor severe disease [18,19]. Thus, it is not clear whether potential gastrointestinal tract infection is protective for intubation or if this is due to reporting bias; it is an area that warrants more rigorous clinical and pre-clinical investigation.

In the current study, the ideal timing for intubation was not defined in CoVID-19, and individual providers could have different thresholds for intubation that would affect which patients experienced our primary outcome. This ideal timing has not been defined in CoVID-19, and individual providers could have different thresholds for intubation. To address this limitation, data on oxygenation status pre-intubation and 72 hours post-intubation was collected. Intubated patients, on average, required 12.9L of oxygen before intubation. Based on the updated and revised version of the “Berlin Definition” of the acute respiratory distress syndrome (ARDS), the intubated patients had, on average, moderate severity acute respiratory distress syndrome on intubation that eventually improved to mild after 72 hours [20]. The average length of intubation was 11.9 days. All of this data shows that despite different intubation patterns, the intubated patients included in this study showed a reasonable level of hypoxemia to warrant intubation.

As a single-centre study, the current findings may lack generalizability. The measurement of C-reactive protein was at a single point at admission, and the change in C-reactive protein over time may be more informative as to the need for intubation. However, the stated goal was to understand the utility of this initial assessment. It is accepted that symptoms were self-reported and may not be a reliable indicator of the full symptomology in the cohort, especially given the rapidity with which some patients were intubated. As reported in patients’ notes, laboratory data and imaging were obtained through routine clinical care, driven by clinical decision making, and have undoubtedly contributed to missing data. Moreover, the final evaluation of the primary endpoint was performed four days following the last admission and may have missed further intubations or clinical changes.

## Conclusion

In patients hospitalised with CoVID-19, high initial C-reactive protein was independently associated with the need for intubation and, in conjunction with high lactate dehydrogenase and *diabetes mellitus*, was strongly predictive of intubation. However, further investigation using larger, multicentre cohort studies are needed to validate the relationship described in this study.
